# Management of behavioral and psychological symptoms of dementia

**DOI:** 10.1192/j.eurpsy.2021.1132

**Published:** 2021-08-13

**Authors:** I. Cruz Da Fonseca, A.M. Romão Franco, R. Mendes, A. Gamito

**Affiliations:** Psiquiatria E Saúde Mental, Centro Hospitalar de Setúbal, Setubal, Portugal

**Keywords:** dementia, BPSD, ECT, management

## Abstract

**Introduction:**

Behavioral and psychological symptoms of dementia (BPSD) are a heterogeneous group of clinical manifestations related to dementia, including apathy, depression, anxiety, delusions, hallucinations, disinhibition, sleep-wake cycle disturbances, aggression and agitation. BPSD have a negative impact on cognitive decline and increase complications.

**Objectives:**

Review treatment management of BPSD including non-pharmacological and pharmacological options, but mainly interventional approaches, such as electroconvulsive therapy (ECT).

**Methods:**

We conducted a search in PubMed and ClinicalKey with the terms: “Behavioral and psychological symptoms of dementia”; “Electroconvulsive therapy”.

**Results:**

The vast majority of patients with dementia will develop one or more BPSD. The etiopathogenesis of BPSD is complex and multifactorial, with multiple direct and indirect factors, namely biological, psychological and social aspects and related to changes in cholinergic, dopaminergic, noradrenergic and serotoninergic circuits. Current guidelines recommend non-pharmacological interventions as the first-line approach for BPSD. Pharmacotherapy is often applied, but it carries out the risk of serious side-effects and pharmacologic interactions. There is now growing evidence that interventional approaches, such as ECT, could be safe and efficient when previous treatment options have been exhausted or ineffective, with few contraindications and transient/limited adverse effects.

**Conclusions:**

BPSD represent a heterogeneous group of non-cognitive symptoms and behavior that affects most of dementia patients. Combination of non-pharmacological and pharmacological interventions is the recommended therapeutic for BPSD. However, there is usually limited clinical improvement and issues related to tolerability and effectiveness. Currently, ECT is considered a safe and effective option.

